# Repurposing propranolol as a drug for the treatment of retinal haemangioblastomas in von Hippel-Lindau disease

**DOI:** 10.1186/s13023-017-0664-7

**Published:** 2017-06-29

**Authors:** Virginia Albiñana, Rosa María Jiménez Escribano, Isabel Soler, Luis Rodríguez Padial, Lucia Recio-Poveda, Karina Villar Gómez de las Heras, Luisa María Botella

**Affiliations:** 1 Centro de Investigaciones Biológicas, CSIC, Madrid, Spain; 2 Hospital Virgen de la Salud (SESCAM), Toledo, Spain; 3 SSCC - Servicio de Salud de Castilla-La Mancha (SESCAM), Toledo, Spain; 4 Centro de Investigación Biomédica en Red de Enfermedades Raras (CIBERER), Madrid, Spain

**Keywords:** von Hippel-Lindau disease (VHL), pVHL, Hypoxia inducible factor, Retinal haemangioblastoma, Juxtapapillary and peripheral haemangioblastoma, Propranolol, Beta-blockers

## Abstract

**Background:**

Von Hippel-Lindau (VHL) disease is a rare oncological disease with an incidence of 1:36,000, and is characterized by the growth of different types of tumours. Haemangioblastomas in the central nervous system (CNS) and retina, renal carcinoma and pheochromocytomas are the most common tumours. The absence of treatment for VHL leads to the need of repeated surgeries as the only option for these patients. Targeting VHL-derived tumours with drugs with reduced side effects is urgent to avoid repeated CNS surgeries. Recent reports have demonstrated that propranolol, a β-blocker used for the treatment of hypertension and other cardiac and neurological diseases, is the best option for infantile hemangioma (IH). Propranolol could be an efficient treatment to control haemangioblastoma growth in VHL disease given its antiangiogenic effects that were recently demonstrated by us. The main objective of the present study was the assessment of the efficacy and safety of propranolol on retinal haemangioblastoma in von Hippel-Lindau disease (VHL).

**Methods:**

7 VHL patients, from different regions of Spain, affected from juxtapapillary or peripheral haemangioblastomas were administered 120 mg propranolol daily. Patients were evaluated every 3 months for 12 months, at Virgen de la Salud Hospital (Toledo). The patients had juxtapapillary or peripheral haemangioblastomas but had refused standard treatments.

**Results:**

Propranolol was initiated with a progressive increase up to a final dose of 120 mg daily. All tumours remained stable, and no new tumours appeared. The reabsorption of retinal exudation was noted in the two patients having exudates. No adverse effects were recorded. VEGF and miRNA 210 levels were monitored in the plasma of patients as possible biomarkers of VHL. These levels decreased in all cases from the first month of treatment.

**Conclusions:**

Although more studies are necessary, the results of this work suggest that propranolol is a drug to be considered in the treatment of VHL patients with retinal haemangioblastomas. VEGF and miRNA 210 could be used as biomarkers of the VHL disease activity.

**Trial registration:**

The study has a clinical trial design and was registered at EU Clinical Trials Register and Spanish Clinical Studies Registry, EudraCT Number: 2014–003671-30. Registered 2 September 2014.

## Background

Von Hippel-Lindau disease is a rare, genetic, hereditary, highly disabling and debilitating disease, which frequently leads to premature death. Von Hippel-Lindau disease is a familiar cancerous disease, with a dominant pattern of inheritance. Its incidence in the population is 1/36,000 [1].

Clinical manifestations include multiple benign and malignant tumours that appear throughout the lifespan of the patient: haemangioblastomas in the central nervous system and retina and cysts and tumours at other levels (serous cystadenoma and pancreatic neuroendocrine tumours, renal clear cell carcinoma and renal cysts, endolymphatic sac tumor, pheochromocytoma and paraganglioma, and cystadenoma of the epididymis and broad ligament) [1, 2].

Retinal haemangioblastomas are typically the most common and earliest presentation of VHL disease [1, 3]^,^. These lesions can occur in about 50% of the VHL patients and mark the debut of the disease in one third of cases. The mean age at diagnosis is 25 years (range: 1–68 years), but these lesions can occur also in infancy [2, 4]. These tumours are often multiple and bilateral, and their size varies from less than one to several optic discs in diameter.

Pathologically identical to the CNS haemangioblastomas [5, 6], these lesions can be classified as peripheral and juxtapapillary (when appear on the optic nerve or near it). Both peripheral and central hemangiomas may cause exudative and tractional retinal detachment, haemorrhages, glaucoma and cataract, leading to blindness.

Unilateral or bilateral amaurosis is relatively frequent among the VHL population due to the development of multiple tumours, which in many cases could be prevented or delayed with proper monitoring and early treatment. When these lesions begin to grow, they are extremely small and difficult to visualize. Generally small lesions can be treated with greater success and fewer complications compared with larger ones [7]. Most peripheral retinal tumours can be treated with laser photocoagulation (small peripheral tumours) or cryotherapy (larger tumours), but these current treatments cannot be used when the tumor is near the optic nerve. In these cases, the most generalized therapeutic approach is only surveillance, given the high risk of damaging the optic nerve [2].

Photodynamic therapy has been used with uneven results [8–12]. Some antiangiogenic drugs, such as bevacizumab and ranibizumab, have been used [13–15], but they do not provide long-term cessation of tumours’ growth [16, 17]. To date no treatment has proven effective in changing the course of the disease, and the possibility that these patients experience improvement when treated with propranolol is undoubtedly an outstanding therapeutic need.

Propranolol hydrochloride is a synthetic β-adrenergic receptor blocking agent marketed for more than 50 years, and whose safety has been largely demonstrated. It has recently been approved for a new indication: the treatment of proliferative infantile haemangioma. This drug has several potential mechanisms of action described in the literature: local haemodynamic effect, antiangiogenic, apoptosis of capillary endothelial cells and reduction of signalling pathways VEGF and bFGF [18, 19].

Previous evidences in the literature demonstrated that propranolol, a drug used to treat arrhythmias, migraines, hypertension and other cardiac and neurological diseases, is effective in the treatment of infantile hemangioma, the most common vascular tumour in new-borns. Its effect was discovered by chance in 2008 [20] and propranolol is the treatment of choice for this vascular tumour.

Propranolol has recently begun to be tested in breast cancer *(* *ClinicalTrials.gov* *Identifier: NCT01847001)* and melanoma *(* *ClinicalTrials.gov* *Identifier: NCT02962947)*, after clinical evidence suggesting that the use of beta-blockers such as propranolol, could increase relapse-free and overall survival [21–24]. The data from previous studies with propranolol to treat angiomas at different levels -, including airway [25, 26] and cerebral cavernous angioma [27–29], support the plausibility of experimental use in this rare disease with a poor prognosis and no pharmacological treatment.

Specifically focusing on retinal pathology, several case reports using propranolol for the treatment of diffuse choroidal haemangiomas typical of Sturge-Weber syndrome have recently been published, with positive results [30].

Given that haemangioblastomas are proliferative vascular tumours, and the natural evolution of papillary and juxtapapillary tumours in VHL disease leads to amaurosis, we hypothesized that propranolol could also function to reduce the growth of those retinal haemangioblastomas where the use of standard treatments could trigger a rapid loss of vision. We propose an experimental use of propranolol as a therapeutic alternative. Based on previous results of propranolol efficiency for IH treatment [18, 31–33] and our own results demonstrating that propranolol acts as antiangiogenic in endothelial cells [34], we hypothesized that propranolol could act by decreasing HIF levels and thereby downregulating the HIF target program. Interestingly, all the HIF target genes, including VEGF (Vascular endothelial growth factor), MMPs (Metalloproteases), EPO (Eritropoietin) or FGF (Fibroblast growth factor), among others, are absolutely necessary for the survival and progression of tumours in general and for haemangioblastomas in particular. Haemangioblastomas are complex tumours consisting of different cellular types, with stromal (undifferentiated mesenchymal cells) and endothelial cells as the main components. When haemangioblastoma cells are subjected to long-term propranolol treatment, haemangioblastoma cells first stop proliferating, and then cell death is detected from the empty spaces in the plates. As explained by previous results, the death must be attributed to apoptosis [35]. Therefore, these results led us to consider the hypothesis that propranolol could be an efficient treatment for haemangioblastomas, through inhibition of HIF and consequently all of its gene targets in highly vascularized tumours, where HIF is constitutively expressed. One of the crucial HIF targeting actions is the angiogenesis process via VEGF. We suggest that propranolol acts therefore by an antiangiogenic mechanism, with VEGF being one of the downregulated genes by the propranolol treatment.

With this background in mind, we hypothesized the following:

- Null hypothesis for the primary endpoint: retinal haemangioblastomas progress in size or complications from baseline value to the end of the treatment period at the 12th month.
- Alternative hypothesis: retinal haemangioblastomas have decreased in size or remain stable compared to baseline, after the completion of the treatment period.

## Methods

We designed an open, pilot clinical trial, to evaluate the effectiveness and safety of propranolol administered over a period of 1 year, for te treatment of patients with von Hippel-Lindau disease and papillary or juxtapapillary haemangioblastoma non eligible for standard treatment (laser or cryotherapy), or peripheral retinal haemangioblastomas for which patients had rejected standard therapies. A total of 7 VHL patients who met the inclusion criteria were recruited. The study included clinic visits at Virgen de la Salud Hospital (Toledo), at baseline and at months 1, 3, 6, 9 and 12 of treatment. A follow-up visit was scheduled after approximately 30 days for patients who were withdrawn from the study. EudraCT Number: 2014–003671-30. Registered 22 September 2014.

The primary endpoint of the study was the measurement of the number and size of haemangioblastomas. The secondary end points were the visual acuity, exudation and exudative retinal detachment. As exploratory objetives, the detection and quantification of plasma biomarckers, VEGF and miRNA 210 and the detection of HIF-controlled targets in the PBLs (peripheral blood leukocytes) of the patients along the trial were performed.

The biomarkers study could serve to check a possible relationship between them and the evolution of the disease. The patients didn’t receive any other treatment which could interfere with the outcomes of the clinical trial.

The Spanish association of patients *Alianza VHL* informed their members of the study during its annual meeting and through the internet, inviting VHL patients with juxtapapillary tumours to participate in the clinical trial.

**Inclusion criteria**: patients diagnosed with von Hippel-Lindau disease presenting ocular involvement due to typical haemangioblastomas appearing in the retina in the course of the disease. Within this population, patients were selected if they met one of two criteria:

1. Papillary or juxtapapillary haemangioblastoma, non-eligible for standard treatment (laser photocoagulation or cryotherapy) due to the high risk of visual loss.
2. Peripheral retinal haemangioblastomas for which patients had rejected standard treatments.

The pharmaceutical form and strength used was propranolol 40 mg, film-coated tablets, 1 every 8 h up to a total dosage of 120 mg/day. At this dose, the safety and tolerability of the drug is acceptable. Nevertheless, an experienced cardiologist in handling propranolol, monitored closely the treatment and the response of patients.

In each visit, blood was extracted for the quantification of molecular biomarkers in the research sub-study.

### VEGF determination in plasma

A Quantikine Human VEGF ELISA kit from R&D Systems (Abingdon, UK) was used to quantitatively determine human VEGF-A concentrations in plasma of the patients included in the study during the different visits from 0 to 12 months of propranolol treatment.

### Real-time RT (RT-qPCR)

Total cellular RNA was extracted from peripheral blood cells (PBLs) from each patient following the clinical trial, using a Nucleo Spin RNA kit (Macherey-Nagel, Düren, Germany). One microgram of total RNA was reverse-transcribed in a final volume of 20 μl with the First Strand cDNA Synthesis Kit (Roche, Mannheim, Germany) using random primers. The SYBR Green PCR system (BioRad, Hercules, CA, USA) was used to perform real-time PCR with an iQ5 system (Biorad, Spain). The sequences of the oligonucleotides used corresponded to the following shown in Table 1:

**Table 1:** Primers used for qPCR amplications

GENE	Forward	Reverse
18S	5′- CTCAACACGGGAAACCTCAC - 3′	5′-CGCTCCACCAACTAAGAACG - 3′.
BAX	5′- CACTCCCGCCACAAAGAT - 3′	5′- CAAGACCAGGGTGGTTGG - 3′
EPO	5′ - TGTTTTCGCACCTACCATCA - 3′	5′ - AAGTCACAGCTTGCCACCT - 3′
SOX2	5′ – GGGGGAATGGACCTTGTATAG - 3′	5′ – CGCTCCACCAACTAAGAACG - 3′
OCT4	5′- CTTCGGATTTCGTCTTCTCG-3′	5′- CTTAGCCAGGTCCGAGGAT - 3′

As an internal control, mRNA levels of 18S were measured. Amplicons were detected using an iQ5 system (BioRad). The samples were assessed in triplicate and the experiment was repeated twice.

### miRNA 210 plasma quantification

Total RNA was isolated from 50 μl of plasma using miRneasy kit (Qiagen) and resuspended in 40 μl. Relative miRNA levels were normalized to one spike-in miRNAs: cel-miR-39, 5′-UCACCGGGUG UAAAUCAGCUUG-3′ (Applied Biosciences).

Kits: sequences for qPCR of human miR 210 and cel-miR39 were ordered from Quantabiosciences. In addition the following kits were used for the reverse transcription and the PCR synthesis: qScript™ microRNA cDNA Synthesis Kit and PerfeCTa ® Universal PCR from Quanta. The procedure followed all the manufacturer instructions.

### Statistics

Given that this is a pilot clinical trial, statistics data were not planned beforehand. Moreover, it is unlikely that we will obtain statistical power for tumour size with only seven patients. However, molecular data on biomarkers were subjected to statistical analysis. The data represent mean ± SD. Differences in mean values were analysed using the Student’s t-test. *P*-values of <0.05 were considered to be statistically significant; statistically significant values are indicated with asterisks (*P < 0.05; **P < 0.01; ***P < 0.005).

## Results

Seven patients volunteered for the study. Some of them decided to participate because their tumours continued progress despite treatment with laser photocoagulation. Four patients had previous severe effects, including impaired vision, as a result of exudation due to active tumours. Two patients had a recent diagnosis of VHL disease. One patient was lost to follow-up because she had to undergo a programmed surgery and did not continue afterwards.

Table 2 shows the age, sex, type of mutation and summary of the main clinical outcomes.

**Table 2:** Genetics and active tumours of the patients along the clinical trial

					First visit				Last visit (12 months later)	
Patient	*Age*	*Gender*	Gene VHL locus	Mutation	Left eye		Right eye			
					*Tumours*	*Exudation*	*Tumours*	*Exudation*	*Tumours* ^*a*^	*Exudation*
1	36	M	Intron 2	Splice g. 8665, A > G; c.DNA 463,+2A > C	1 peripheral	-	1 peripheral	Great exudation	No changes	No exudation
2	33	F	Intron 2	Splice.g.8665, A > G; c.DNA 463,+2A > C	1 juxtapapillary	-	-	-	No changes	-
3	50	F	Exon 3	c.501 A > T	1 juxtapapillary	Exudation	-	-	No changes	Much less exudation
4	20	M	Exon 3	c.501 A > T	1 juxtapapillary	-	-	-	No changes	-
5	15	M	Ex1–2-3	del complete VHL	-	-	1 peripheral	-	No changes	-
6	38	F	Exon 1	del exon 1	1 juxtapapillary	-	-	-	*Withdrawn* ^*b*^	-
7	22	M	Exon 3	c.486, C > G	2 peripheral	-	2 peripheral		No changes	-

This table exclusively presents active tumours in the initial or final evaluation, which were the principal objective of treatment

^a^“No changes” indicates no more tumours, or growth of previous tumours

^b^Withdrawn from the clinical trial due to a scheduled surgery

All patients visited the Ophthalmology Department at Virgen de la Salud Hospital after 1 month, and then, every 3 months. The Ophthalmologist researcher took pictures of the affected retina in each visit.

**As the main clinical outcomes (**Table 1), the number and size of all the tumours present on the retina, in all patients, remained stable during the clinical trial, without any other treatment, regardless of propranolol administration. Tumor size was measured at the beginning and at each visit (data not shown). No significant changes in size were noted. However, no new tumours appeared during the follow-up period. The most remarkable result was the reabsorption of exudates in the 2 patients with retinal exudation. The disappearance of the lesion was progressive and clear. The patients did not receive any other treatment that could interfere with the outcomes of our clinical trial.

Of note, without treatment, and occasionally with treatment, retinal haemangioblastomas can continue to grow and affect visual function by causing an exudative retinal detachment [7]. The only method to reduce the exudation is laser coagulation or cryotherapy of tumour feeding vessels, if possible.

The only side effect observed was low blood pressure in patient 4. In this case, the dosage of 120 mg/day was achieved more slowly, with a progressive increase over more weeks compared with other patients, under cardiologist control. Hypotension is a well-known and frequent side effect of beta-blockers, as propranolol, which can be partially prevented by increasing the dose slowly.

### Data from the clinical registries

**Fig. 1:**
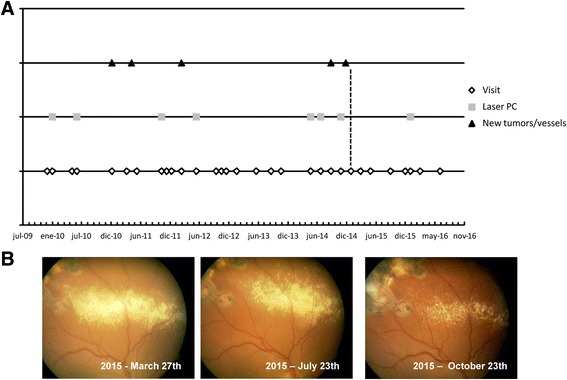
**a** Evolution of Patient 1 included in the clinical trial. The graphic is based on the notes written by the different ophthalmologists in his clinical history, and represents the evolution before and during the clinical trial. The *dotted lines* denote the time of propranolol administration in the clinical trial. **b** The patient had retinal exudation that decreased as treatment progressed, as demonstrated at three different time points during the clinical trial

**Fig. 2:**
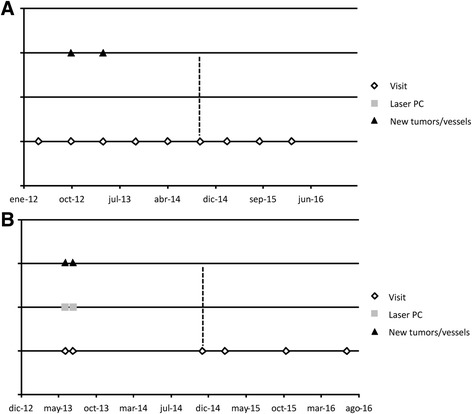
Evolution of Patients 2 and 5 included in the clinical trial. **a** Patient 2: the graphic is based on the notes written by the different ophthalmologists in his clinical history, and represents the evolution before and during the clinical trial. The *dotted lines* denote the time of propranolol administration in the clinical trial. **b** Patient 5: the graphic is based on the notes written by the different ophthalmologists in his clinical history, and represents the evolution before and during the clinical trial. The *dotted lines* denote the time of propranolol administration in the clinical trial

**Fig. 3:**
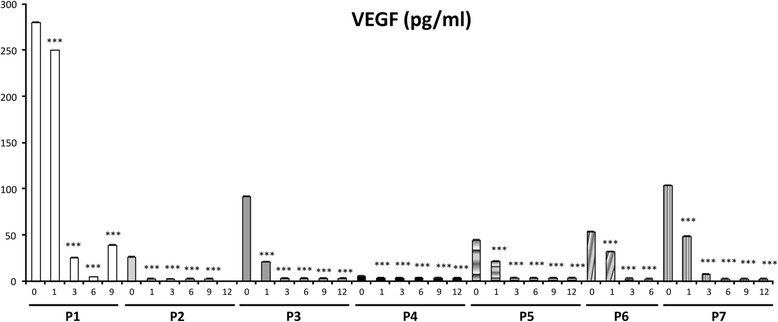
Evolution of VEGF plasma levels of the different patients (P1-P7) at the different visits during the clinical trial. Arrows indicate three cases wherein VEGF levels were initially greater than the normal threshold, and the decrease was significant, reaching normal levels following propranolol treatment

In addition to the controls performed by the ophthalmologist researcher (Toledo), the patients continued with their usual controls suggested by their ophthalmologists at their place of residence. This feature helped us to be more objective with the interpretation of our results. The clinical registries of patients before the clinical trial help to make the outcomes of this trial significant. The evolution of the registries of some of the patients are shown in Figs. 1 and 2. Fig. 1a shows the evolution of patient 1 in a graphic based on the notes written by ophthalmologists in his clinical history. This patient had a clinical register with multiple tumours, treated by multiple photocoagulations. The dotted lines denote the time under the clinical trial with propranolol treatment. Ophthalmologist wrote in his clinical history “no new lesions/no active lesions” during the visits he attended from January 2015 until December 2015. Moreover, he had retinal exudation which decreased during the months of treatment (Fig. 1b). He had also the most significant reduction in ***VEGF levels*** during the study, as shown in Fig. 3.

Fig. 2a presents the evolution of patient 2. She had several tumours that remained stable in number and size. Fig. 2b shows evolution of patient 5. The yellow line indicates the start of treatment with propranolol. Patient 6 had a severe retinal disease with important visual impairment. She had to undergo retinal surgery, scheduled before the start of the trial, and was admitted in case treatment could avoid the surgery, but finally, as it was necessary, the patient withdrew from the clinical trial.

### Biomarkers used in the clinical trial as indicators of prognosis

Three types of biomarkers from blood samples have been used for the patient follow-up: VEGF plasma levels, given that VEGF is a direct HIF target; qPCR with other HIF target genes pertinent to the development of haemangioblastomas: *Epo*, *Sox-2* and *Oct-4*; and the pro-apoptotic gene *Bax*.

Finally, as part of an innovative technique, we analysed the *miR210* levels in plasma. *Mir210* is a micro-RNA responsive to hypoxia that we have used as a new putative biomarker to follow VHL patient evolution during the clinical trial.

### VEGF as a biomarker in circulating plasma

As a marker of angiogenesis related to VHL, and HIF target, VEGF was measured by ELISA in the plasma of all the patients, before they started the clinical trial, and every time they visited the researcher ophthalmologist. In all patients, VEGF levels decreased from the first month of treatment. (P *= Patient)* in a significant manner *(p < 0.001*), reaching normal levels (concentration < 50 pg/ml) in all cases after 3 months of treatment (Fig. 3).

### qPCR of HIF targets and *Bax* proapoptotic gene during propranolol treatment

**Fig. 4:**
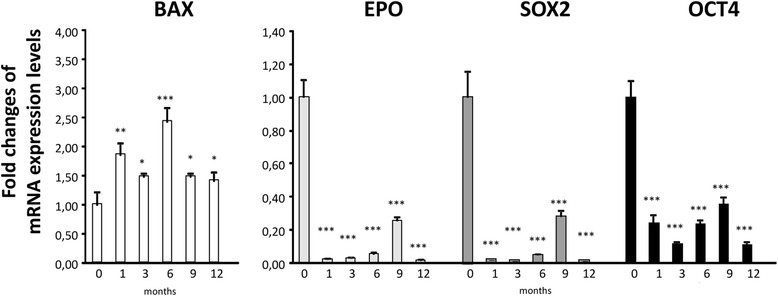
RT-PCR results of the relative expression levels of different HIF target genes normalized to 0, following propranolol treatment. The proapoptotic gene *Bax*, is upregulated relative to the initial time point during the clinical trial, whereas *Epo*, *Sox,* and *Oct 4* gene expression is downregulated in response to propranolol. The results are representative of one of the patients included in the clinical trial. RNA was obtained from the PBL fraction. Each RT-PCR was repeated at least three times and in triplicate

The mRNA expression levels of other HIF-target genes, *Epo, Sox-*2 and *Oct4* (genes triggered by HIF-1 at transcription level), and the pro-apoptotic gene *Bax* (repressed by the β-adrenergic pathway) were also assessed by qRT-PCR in the cells of the PBL fraction from each patient during the clinical trial. Fig. 4 shows reveals a 1.5 to 2.5 fold increase in the *Bax* expression and reduced *Epo*, *Sox-2* and *Oct4* (genes involved in angiogenesis, and stemness, respectively) expression after propranolol treatment. The graphs are from a representative patient. The results are compatible with a situation wherein the existing haemangioblastomas should not proliferate due to decrease in proangiogenic genes (*Epo and VEGF*), a decrease in the expression of tumour progression genes, *Sox-2 and Oct4* and an increase in proapoptotic *Bax*. All cells in patients are heterozygous for the *Vhl* gene, with the exception of cells from haemangioblastomas (*Vhl* ^*−/−*^). The response to propranolol treatment is systemic and depending on the β_2_ adrenergic receptors expressed by the cells in the organism. It is noteworthy to mention that in three cases when blood cell counts were available before and after the clinical trial, a decrease of total leukocytes number was detected, but keeping always values over 4.000 leukocytes/ml within normal range. Moreover, an abnormal number of reticulocytes detected in one of these patients before the clinical trial completely disappeared after propranolol treatment. These data are in agreement with the increase of *Bax* during the clinical trial.

### Evolution of *mir210*

Mir 210 was used as a hypoxia microRNA target induced by HIF [36] and therefore could be a good marker in parallel with other HIF targets, including *Epo, Sox-2* and *Oct4;* VEGF in plasma and *Bax* for monitoring the evolution of the patients in the clinical trial.

**Fig. 5:**
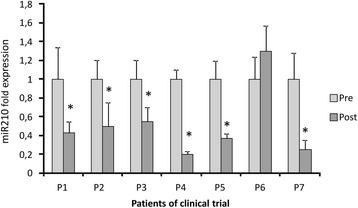
RT-PCR results of the *miR210* gene as measured from plasma samples of the patients included in the clinical trial. The quantification of miR210 before and after 1/3 months (Pre/Post) of propranolol treatment is presented. All the decreases are significant at *p* < 0.001

As observed in Fig. 5, miR210 was reduced at the beginning of propranolol treatment. Similar results were noted for VEGF, with the exception of P5 where there was no significant change. After 3 months of propranolol treatment, VEGF was significantly reduced, and *miR210* levels were reduced by half (P2–3 m).

## Discussion

The present manuscript shows the results of a pilot clinical trial, with 120 mg/day of propranolol to delay/stop the growth of retinal haemangioblastomas in VHL patients. The fact that the number and size of all the tumours present on the retina at the beginning of the clinical trial remained stable without any other treatment, other than propranolol is quite promising, especially when compared with the previous evolution of these patients. One could argue that this result could be attributable to the natural behaviour of the retinal haemangioblastomas which might be quiescent for this period. However, the progressive and clear reabsorption of exudation cannot be explained by tumour quiescence. The introduction of molecular biomarkers to follow the patient’s evolution in parallel helped to reinforce the clinical observations. The concomitant reduction of vascular endothelial growth factor (VEGF) plasma levels, almost from the beginning of the treatment is a clear change that is likely attributable to propranolol.

As a marker used for the first time in von Hippel-Lindau disease, we have introduced the micro- RNA *miR210*, which is a direct target of HIF [36]. The results of plasma *miR210* detection during the clinical trial, reveal an evolution parallel to VEGF. Therefore, in this context, we could consider plasma levels of VEGF and *miRNA 210* as biomarkers for VHL disease and especially suggestive of bona fide *biomarkers* for good prognosis in the evolution of the retinal disease.

If we attempt to correlate VEGF/miR210 levels with the clinical findings before the start of the treatment, we realize that the two highest VEGF values in P1 and P3 (280 pg/mL and 120 pg/mL, respectively), correspond to the two patients harbouring haemangioblastomas with exudates. These patients exhibited normalized VEGF levels, after 3 and 1 month, respectively, and miR210 levels were reduced by 60% and 50%. The reabsorption of exudates began after 3 months, and was almost complete after 6 months. Therefore, the correspondence between VEGF/miR210 levels and the clinical outcomes suggest that these are good VHL biomarkers and further support their use as good therapeutic monitors during propranolol treatment.

**Fig. 6:**
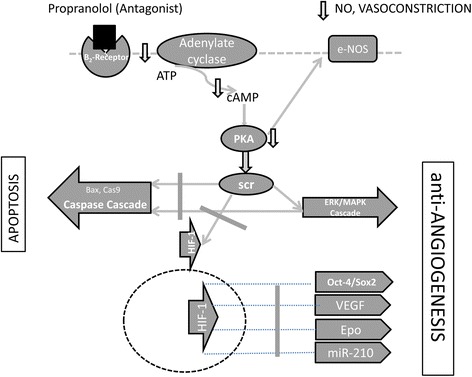
Hypothesis summarizing the propranolol mechanism involved in haemangioblastoma cells. As a β-blocker, propranolol would reverse three main targets promoting the cessation of division and apoptosis, anti-angiogenesis, and vasoconstriction

In vitro results obtained in our lab, suggest that propranolol decreases HIF levels in haemangioblastoma cell. Thus, the HIF targets, are at least, partially silenced/decreased [35]. Consequently, in the absence of essential factors for survival (those HIF targets), and due to the pro-apoptotic effect of propranolol [18, 34, 35] the tumour cells stop dividing. As a β-blocker, propranolol reverses three main targets: halting division and triggering apoptosis, inducing anti-angiogenesis, and promoting vasoconstriction. Thus, on one hand, propranolol may stop HIF-inducible functions, such as VEGF-, Epo-, Sox-2- and Oct-4- dependent growth of haemangioblastomas [4, 37], and inhibition of angiogenesis. On the other hand, propranolol promotes apoptosis by inducing Bax and the caspase cascade. These two facts together may contribute to the control of haemangioblastoma growth. The expression of these genes was also measured during the clinical trial, supporting the rationale of propranolol’s way of action and clinical effects. In particular, the influence of propranolol on apoptosis, likely explains the role of adrenergic antagonists in the pathogenesis and therapy of inflammation, cardiovascular diseases, and bronchial asthma [38]. In addition, the total leukocyte count in those patients with blood tests available were decreased but keeping the normal range over 4.000 leukocytes/ml. Abnormal number of reticulocytes disappeared also during propranolol treatment in one patient. This is in agreement with the influence of propranolol in apoptosis. Therefore total counts of blood cells should be monitored in patients under treatment, to detect changes. Fig. 6 illustrates the pathways blocked by propranolol via its action as an antagonist of β2 ligands.

Although the clinical trial is now complete, we have planned to continue the follow -up of the six patients. Among them, 4 patients have decided to continue taking the drug as a compassionate use of a drug under investigation. These data will be useful to establish the effect on the retina during a long-term use. Two patients decided to abandon the treatment. We will also follow-up these patients to collect data of their evolution too (before and after study).

Given that some recent publications suggest that the optimal dosage of propranolol for a complete recession of infantile haemangioma is around 3 mg/kg [39], this dose should be equivalent to the highest dose used in our in vitro experiments (100 μM) [34]. Thus, we have consulted with our ophthalmologist and cardiologist about the possibility of initiating another clinical trial with a higher dose of propranolol, closer to 3 mg/kg of body weight, including more patients and with fewer previous interventionist treatments. It would also be advisable to have a longer follow-up of patients, of at least 3 years.

Due to the excellent response of the retinal exudation to the treatment with propranolol, more patients with this condition should be included, to confirm these initial results in the present clinical trial.

The results of the clinical trial, together with the previous in vitro knowledge generated in our first publication of OJRD [34] led to the recent orphan drug designation of propranolol by EMA to treat von Hippel Lindau disease EU/3/17/1841.

## Conclusions

The fact that all the retinal tumours remained stable and no new tumours appeared during the follow-up period, without any other treatment but propranolol suggests that propranolol is a promising therapeutic drug for retinal haemangioblastomas, and perhaps for other ocular pathologies with retinal exudation and high VEGF levels (as macular degeneration). It would be convenient to explore the use of higher dosages (i.e., closer to 3 mg/kg body weight/day).

The results of the clinical trial, together with the previous publication in OJRD [35] led to the orphan drug designation of propranolol by EMA to treat von Hippel Lindau disease **EU/3/17/1841**.

The evolution of VEGF and *miRNA 210* in all the patients throughout the study, which paralleled the inactivity of the retinal disease, suggest that they may be useful as biomarkers of VHL disease activity. To the best of our knowledge, these are the first biomarkers described in the literature to monitor the VHL disease activity.

## Abbreviations

CNS: Central nervous system
FBS: Foetal bovine serum
FGF: Fibroblast growth factor
HIF: Hypoxia inducible factor
HRE: Hypoxia responsive element
IH: Infantile hemangioma
MMP: Matrix metalloproteinases
PBLs: Peripheral blood leukocytes
pVHL: Von Hippel Lindau protein
RPMI: Roswell Park Memorial Institute
RT-PCR: Reverse transcription polymerase chain reaction
SDS-PAGE: Sodium dodecyl sulfatepolyacrylamide gel electrophoresis
SOX-2 or SRY-box 2: Sex-determining region Y-box
VEGF: Vascular endothelial growth factor
VHL: Von Hippel-Lindau
